# Repercussions of moving patients on the context of practice: perspectives of the nursing team

**DOI:** 10.1590/1518-8345.7042.4113

**Published:** 2024-03-15

**Authors:** Mariana Santos de Campos, Danielle Fabiana Cucolo, Marcia Galan Perroca

**Affiliations:** ^1^ Faculdade de Medicina de São José do Rio Preto, São José do Rio Preto, SP, Brazil.; ^2^ Pontifícia Universidade Católica de Campinas, Campinas, SP, Brazil.

**Keywords:** Patient Transfer, Workflow, Nursing Staff, Workload, Health Care Process Assessment, Practice Management, Transferencia de Pacientes, Flujo de Trabajo, Personal de Enfermería, Carga de Trabajo, Evaluación de Procesos, Atención de Salud, Gestión de la Práctica Profesional, Transferência de Pacientes, Fluxo de Trabalho, Recursos Humanos de Enfermagem, Carga de Trabalho, Avaliação de Processos em Cuidados de Saúde, Gerenciamento da Prática Profissional

## Abstract

**Objective:**

to examine the nursing team’s view of the repercussions of moving patients (admissions, transfers and discharges) on the organization of work and the delivery of care.

**Method:**

this is a qualitative study using the focus group technique, conducted with 23 professionals - 12 nurses, eight nurse technicians and three nurse assistants working in three inpatient units at a teaching hospital in the countryside of Sao Paulo. Four meetings took place between November 2021 and March 2022. The reports were analyzed thematically using MAXQDA software.

**Results:**

two thematic categories emerged: the influence of structural factors and work organization on the intra-hospital moving of patients; it demands time, generates work overload and interferes with the delivery of care.

**Conclusion:**

the volume of moving patient associated with unforeseen demands, care complexity and insufficient staff and resources have a negative impact on the delivery of care, with clinical risks and work overload. The findings make it possible to improve the regulation of patients entering and leaving the units, work organization and care management, avoiding clinical risks, delays, omissions and work overload.

## Resumen

**Objetivo:**

examinar la perspectiva del equipo de enfermería sobre las repercusiones del movimiento de pacientes (admisiones, traslados y altas) en la organización del trabajo y en la prestación de cuidados.

**Método:**

estudio cualitativo utilizando la técnica de grupo focal llevado a cabo con 23 profesionales, incluyendo 12 enfermeros, ocho técnicos y tres auxiliares de enfermería de tres unidades de internación de un hospital universitario del interior de São Paulo. Se realizaron cuatro encuentros entre noviembre de 2021 y marzo de 2022. Los relatos fueron analizados en la modalidad temática con la ayuda del *software* MAXQDA.

**Resultados:**

surgieron dos categorías temáticas: la influencia de factores estructurales y de la organización del trabajo en el movimiento intrahospitalario de pacientes; y, el movimiento de pacientes demanda tiempo, genera sobrecarga de trabajo e interfiere en la prestación de cuidados.

**Conclusión:**

el volumen de movimientos de pacientes asociado a demandas imprevistas, complejidad asistencial e insuficiencia de personal y de recursos repercute negativamente en la prestación del cuidado con riesgos clínicos y sobrecarga laboral. Los hallazgos permiten mejorar la regulación de las entradas y salidas de pacientes en las unidades, la organización del trabajo y la gestión del cuidado, evitando riesgos clínicos, retrasos, omisiones y sobrecarga laboral.

## Resumo

**Objetivo:**

examinar o olhar da equipe de enfermagem quanto às repercussões da movimentação de pacientes (admissões, transferências e altas) na organização do trabalho e na entrega do cuidado.

**Método:**

estudo qualitativo utilizando técnica de grupo focal conduzido junto a vinte e três profissionais sendo doze enfermeiros, oito técnicos e três auxiliares de enfermagem lotados em três unidades de internação de um hospital de ensino do interior de São Paulo. Ocorreram quatro encontros, entre novembro de 2021 e março de 2022. Os relatos foram analisados na modalidade temática com o auxílio do *software* MAXQDA.

**Resultados:**

emergiram duas categorias temáticas: a influência de fatores estruturais e da organização do trabalho na movimentação intra-hospitalar de pacientes; e a movimentação de pacientes demanda tempo, gera sobrecarga de trabalho e interfere na entrega do cuidado.

**Conclusão:**

o volume de movimentações de pacientes associado às demandas imprevistas, complexidade assistencial e insuficiência de pessoal e de recursos repercutem negativamente na entrega do cuidar com riscos clínicos e sobrecarga laboral. Os achados possibilitam aprimorar a regulação das entradas e saídas de pacientes nas unidades, organização do trabalho e gestão do cuidado evitando-se riscos clínicos, atrasos, omissões e sobrecarga laboral.

**Highlights:**

**(1)** Moving patients around the hospital requires structure and work organization.

**(2)** Nursing estimates dedicating 10-15 minutes to 2-3 hours of work on these interventions.

**(3)** Frequency, unpredictability and complexity of care have a negative impact on care.

**(4)** Unfavorable conditions for moving generate care and occupational risks.

## Introduction

 Moving patients intra-hospital occurs frequently in hospital practice and can be understood as a transition process motivated by clinical (health and disease process) and/or organizational issues ^(^ ^1^ ^)^ . It includes events such as admissions, discharges and transfers within and between units. 

 These transitions can be favored or hindered by personal, social and community conditions ^(^ ^1^ ^)^ . From a clinical perspective, the complexity and quantity of moving of patients to be carried out are associated with the availability of staff and adequate materials/equipment to provide care. When teams become overloaded, they are exposed to the omission of information and care, as well as diagnostic and therapeutic errors or delays ^(^ ^2^ ^)^ . 

 In the context of healthcare organizations, the implementation and management of protocols, a culture of teamwork, technological advances, institutional policies for bed occupancy and the use of resources all contribute to successful moving of patients ^(^ ^3^ ^)^ . Interprofessional communication guided by a standardized and mutually agreed tool is another determining factor in the qualification of this process ^(^ ^4^ ^-^ ^5^ ^)^ . Care in patient transportation and health education actions were also identified as contributing elements ^(^ ^5^ ^)^ . 

 Recognizing the logistical network and the clinical demands that drive the flow of inpatients is also highly relevant for controlling healthcare-related infections and for managing the capacity and resolutiveness of hospitals in the care network ^(^ ^6^ ^-^ ^7^ ^)^ . 

 Every day, nurses are challenged to recognize and develop interventions for safe transitions ^(^ ^8^ ^)^ , in particular, motivated by the patient’s clinical condition and by other typologies (situational, developmental or organizational) mobilizing conditioning factors and results ^(^ ^1^ ^)^ . 

 A Swiss study found that the number of admissions, discharges and transfers and the simultaneous occurrence of these demands affect nurses’ workload (NW) ^(^ ^9^ ^)^ . Recent research with Italian nurses confirms this predictive relationship between in-hospital patient transfer and NW ^(^ ^10^ ^)^ . And, as a consequence, a higher risk of adverse events ^(^ ^11^ ^)^ . 

 In the Brazilian context, researchers have identified that nurses can dedicate between 19.3% and 29% ^(^ ^12^ ^)^ of working time to transfers and between 16.3% and 31.5% to hospital admissions ^(^ ^13^ ^)^ , but the national literature on the subject is still scarce. This justifies the development of studies in Brazilian hospitals exploring the moving of patients and the interference(s) with nursing work. 

This research was guided by the question: how does the moving of patients in and out of hospital units influence the practice of nursing professionals? Investigating these demands can contribute to (re)organizing the work and planning of the nursing team in hospital units. It aims to examine the nursing team’s view of the repercussions of moving of patients (admissions, transfers and discharges) on the organization of work and the delivery of care.

## Method

### Study design

 This qualitative research used the focus group (FG) technique to explore the intra-hospital moving of patients, based on Transitions Theory ^(^ ^1^ ^)^ . This theoretical framework encompasses both process and time elements and interpersonal and environmental experiences, and characterizes transitions as a positive event, but one that can also be anchored in (dis)continuity. It is based on four pillars: the nature of transitions (type, pattern and property); personal, social and community conditioning factors; interventions and response patterns (results) ^(^ ^1^ ^)^ . Therefore, the admission, discharge and transfer of patients within hospitals require nursing interventions in order to provide effective care before and/or after the period of change. 

 The Consolidated Criteria for Reporting Qualitative Research (COREQ) guidelines guided the design and presentation of this investigation ^(^ ^14^ ^)^ . 

### Scenario and data collection period

The study was carried out in three inpatient units: a medical clinic (28 beds), a surgical clinic (26 beds) and a medical-surgical unit (45 beds) at an extra-capacity teaching hospital located in the countryside of the state of São Paulo, Brazil. The high-complexity institution, with national health accreditation (level 3), is a reference center for municipalities in the region and also receives users from other states.

The units receive patients from the Unified Health System (SUS) with chronic-degenerative diseases and the specialties of geriatrics, nephrology, gastrology and general surgery. They are staffed by 30 nurses and 80 nursing assistants and technicians. The GFs took place in November 2021 and March 2022.

### Participants and selection criteria

Members of the nursing team (nurses - N, technicians - NT and assistants - NA) were nominated by the nursing managers of the units and/or the institution’s continuing education nurse. To make up the convenience sample, they had to have been with the institution for at least three months (nursing staff) and work in direct patient care (nurses); those on leave (licenses and vacations) were excluded.

Of the 63 professionals (N-28, NT-23, NA-12) invited, 30 agreed to take part in the study. Of these, 23 (N-12, NT-8, NA-3) made up the FG.

### Instrument and procedures for data collection

 Four FG sessions were held with the participation of around six nurses, four technicians and one or two nursing assistants per group. To analyze and determine gaps ^(^ ^15^ ^)^ , as well as identifying the understanding and relevance of the questions, favoring the refinement of the process and readjustment to the proposed objectives ^(^ ^16^ ^)^ , two pilot FGs (PS) were conducted. They were made up of seven professionals, four of whom were nurses (PS 1), two technicians and one nursing assistant (PS 2). Due to the existing hierarchical relationship, the professional categories (N, NT/NA) were separated for the groups. This segmentation criterion avoids professional dominance, allowing for better participation and interaction between members ^(^ ^17^ ^)^ . 

 The invitation to the PS took place online, using a messaging app (WhatsApp ^®^ ), due to the COVID-19 pandemic. At this point, the aim of the research, the availability of dates and times were highlighted and participation was confirmed. Beforehand, the Informed Consent Form (ICF) and the questionnaire with sociodemographic and work-related variables were sent using the survey management application (Google ^®^ Forms). 

 The meetings for the pilot FGs took place remotely via a digital video conference communication platform (Google ^®^ Meet) around the guiding question “In your perception, does the moving of patients, represented by the number of admissions, transfers and discharges in the unit where you work, interfere with: the organization of your work during the shift? On the quality of care provided to patients? In what way?”. This was followed by other questions about the desirable/ideal conditions for performing the job with quality and safety, and unfavorable and extremely unfavorable situations with risks to patients and health professionals. As well as expressing their experiences on the subject, the participants had the opportunity to add aspects that had not been discussed. 

Although the professionals considered the questions to be appropriate and understandable, after analyzing the PS reports, there was a high frequency of mention of the time factor and its impact on work dynamics. Therefore, were included questions about the average time spent per work shift and about carrying out planned activities when moving patients around the units.

With the improvement in the pandemic situation and in view of the findings from the PS, it was possible to schedule the FGs in person, in a private room at the institution, during the daytime. The invitation was sent to the units investigated with the help of the nursing managers and the nurse responsible for continuing education at the institution.

At the beginning of the session, the ICF and the professional profile questionnaire were distributed and, after consenting, the proposed questions were read out. During the meetings, the researcher encouraged the participation of the professionals, explained the points covered in the questions and allowed them to discuss other experiences related to the subject.

All the sessions, including those on PS, were conducted by a researcher with a specialization in management and trained in conducting FGs. With prior authorization, the meetings were recorded using an electronic device and lasted an average of 40 minutes.

### Data treatment and analysis

 Speeches were transcribed in full, exported to MAXQDA software version 2020 ^(^ ^18^ ^)^ and explored according to the thematic modality ^(^ ^19^ ^)^ . During the pre-analysis, the material was organized and read fluidly. Two researchers independently and consensually selected the coding units after exporting them to MAXQDA. The codes were defined after in-depth reading of the reports, in order to compose the categories by aggregating the findings (exploration of the material). The codes and themes were then validated by a third researcher. MAXQDA software was used to generate a word cloud with the codes identified. Finally, interpretations and inferences were made to treat the results from the FGs in line with the theoretical framework adopted ^(^ ^1^ ^)^ . 

To maintain anonymity, the professionals were coded followed by their professional category (Nurse - N; Nursing Assistant and Technician - NA/NT) and ordinal number. This gave rise to acronyms such as FGN1 and FGNT/NA1, sequentially.

### Ethical aspects

The study was approved by the Research Ethics Committee of the São José do Rio Preto Medical School (FAMERP), CAAE No. 47183321.7.0000.5415. Ethical criteria were respected in accordance with the recommendations of Resolution 466/2012 of the National Health Council.

## Results

 The FGs were made up of 12 nurses and eight technicians and three nursing assistants, mostly female, aged between 22 and 49. The average time the nurses had been working was 9.5 (SD=6.4; range 3 to 17) years and the technicians/nursing assistants 8.5 (SD=6.7; range 4 to 21) years. Figure 1 shows other work characteristics of the participants. 

**Figure 2:**
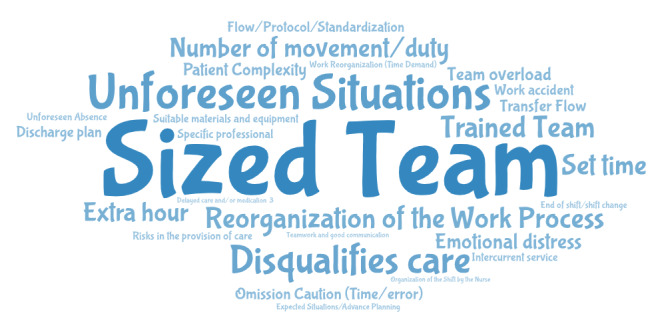
- Cloud of codes extracted from focus groups with nurses and nursing technicians/assistants. São José do Rio Preto, SP, Brazil, 2022

 The coding units that emerged from the FG were grouped using MAXQDA software and are shown in Figure 2 . 

**Figure 1:** - Distribution of focus group participants (n=23) by work unit and shift. São José do Rio Preto, SP, Brazil, 2022

**Category**	**Unit**	**Shift**
N ^*^ 1 (FG ^†^ 1)	MSU ^‡^	Morning
N ^*^ 2 (FG ^†^ 1)	CSU ^§^	Afternoon
N ^*^ 3 (FG ^†^ 1)	CMU ^||^	Afternoon
N ^*^ 4 (FG ^†^ 1)	MSU ^‡^	Morning
N ^*^ 5 (FG ^†^ 1)	MSU ^‡^	Morning
N ^*^ 6 (FG ^†^ 1)	CMU ^||^	Morning
N ^*^ 7 (FG ^†^ 3)	CSU ^§^	Afternoon
N ^*^ 8 (FG ^†^ 3)	MSU ^‡^	Night
N ^*^ 9 (FG ^†^ 3)	CSU ^§^	Afternoon
N ^*^ 10 (FG ^†^ 3)	CSU ^§^	Afternoon
N ^*^ 11 (FG ^††^ 3)	CMU ^||^	Afternoon
N ^*^ 12 (FG ^†^ 3)	CMU ^||^	Night
NT ^¶^ 1 (FG ^†^ 2)	CSU ^§^	Morning
NT ^¶^ 2 (FG ^†^ 2)	CMU ^||^	Night
NT ^¶^ 3 (FG ^†^ 2)	MSU ^‡^	Morning
NT ^¶^ 4 (FG ^†^ 2)	CMU ^||^	Morning
NT ^¶^ 5 (FG ^†^ 4)	MSU ^‡^	Afternoon
NT ^¶^ 6 (FG ^†^ 4)	MSU ^‡^	Afternoon
NT ^¶^ 7 (FG ^†^ 4)	CSU ^§^	Afternoon
NT ^¶^ 8 (FG ^†^ 4)	CMU ^||^	Night
NA ^**^ 1 (FG ^†^ 2)	MSU ^‡^	Afternoon
NA ^**^ 2 (FG ^†^ 2)	CMU ^||^	Night
NA ^**^ 3 (FG ^†^ 4)	CMU ^||^	Afternoon

^*^ N = Nurses;

^†^ FG = Focus group;

^‡^ MSU = Medical-surgical unit;

^§^ CSU = Clinical-surgical unit;

^||^ CMU = Clinical-medical unit;

^¶^ NT = Nursing technician; **NA = Nursing assistant

After analyzing the reports of the nursing team in the FGs, it was possible to extract two thematic categories, each of which was made up of two subcategories: (1) The influence of structural factors and work organization on the intra-hospital moving of patients: factors favorable to the moving of patients, and unfavorable and extremely unfavorable conditions; (2) The moving of patients demands time, generates work overload and interferes with the delivery of care.

### The influence of structural factors and work organization on moving of intra-hospital patient

#### Factors favoring moving of patients

The nurses highlighted the need to standardize the admission, discharge and transfer processes. The implementation of flows with delimited schedules, advance planning of transfers and the use of instruments to guide the transition of care were mentioned as contributory elements in the organization of nursing work.

*(...) if I could be informed at the beginning of each shift, I think that would be ideal, so I could include it in the routine. (...).* (FGN9) 

*(...) try to concentrate on a fixed schedule (...) probable discharges would be decided by a certain time, this would facilitate the creation of a process, organization of care and roles (...) there should be a standard operating procedure for each type of transfer (...).* (FGN12) 

*(...) establish a standardized process (...) mandatory items to be carried out before and after, like a checklist (...)* (FGN1). 

One of the participants’ biggest concerns was the adequate size and training of the nursing team, as well as the availability of sufficient material resources and equipment to meet the health needs of patients considering successful interventions.

*(...) trained staff, effective education (...) as well as a minimum of suitable materials and equipment (...)* . (FGN2) 

*(...) the main thing is an adequate number of staff, where the patient division is not high, so I can provide quality care (...).* (FGNT5) 

Two technicians/nursing assistants pointed to the planning carried out by the nurse and the work organization model based on collaboration between team members as facilitating aspects in the face of care demands, including patient transfers.

*(...) if you’re well organized, if you manage to organize everything, it doesn’t interfere (...) if the nurse manages to organize the patients well (...) our shift is better (...)* . (FGNT5) 

*In my ward, I don’t think this happens much, one always helps the other, most of these activities are organized (...) our team tries to be united (...).* (FGNT7) 

Still in the context of practice, the availability of a support service with trained professionals to transport patients, especially to units with a high number of moving of patients and working different shifts, was mentioned as another issue favorable to the process.

*There should be a team just for these processes, someone to transfer and tidy up the paperwork (...).* (FGN8) 

*(...) if there are transport professionals (...) they are also few in number, even at night there are no (...).* (FGNT2) 

#### Unfavorable and extremely unfavorable conditions

The volume of admissions, transfers and discharges of patients, complications during the shift and the lack of prior communication were considered risk factors in the provision of nursing care.

*(...) when the volume (...) of admissions, discharges and transfers is high during the shift, the rest of the activities are compromised due to the reduced time and this ends up being very bad.* (FGN12) 

*(...) with many patients coming and going, the risks increase for everyone.* (FGNA1) 

*On duty with intercurrences, a destabilized patient in the unit, it becomes very rushed and the risks increase for everyone (...)* . (FGNT2) 

*When urgent and emergency care is provided in the unit, the focus is on the intercurrence.* (FGN4) 

*When discharge has not been planned or communicated beforehand (...) it can be rushed, without attention and this situation is unfavorable to the patient* . (FGN12) 

*(...) an admission arrives without anyone knowing, with no bed arranged, this is terrible (...) communication should be better (...)* . (FGNA3) 

The team also highlighted, as adverse conditions, the insufficient number of staff and equipment/materials, as well as moving of patients that occur at the time of changing shifts and transfers of critically ill patients to intensive care units.

*(...) when the number of nursing staff is inadequate, the quality of care is compromised (...)* . (FGN10). 

*(...) when there is a lack of inputs (...) suitable equipment for the transfer (...).* (FGN2) 

*Unsafe transportation, with a stretcher, makeshift chair, without proper transportation, these are severe risks.* (FGNT1) 

*(...) the risk increases if things happen mainly at times of overload and close to shift changes (...) if there is an admission at that time, it will certainly be done more quickly and with less attention (...).* (FGN4) 

*The moment we are with a patient who has to be transferred to the ICU (...).* (FGNT5) 

Structural factors, such as the size of the team, material resources/equipment and support/transport services, clinical complexity and the frequency of patient transfers, can influence the care provided by nurses during admissions, discharges and transfers. Collaborative work based on standardized flows and processes can promote the practice of nursing.

### Moving patients takes time, generates work overload and interferes with the delivery of care

#### Quality and safety in health care

Nurses show great concern for occupational safety when moving patients without the necessary professional staff and in situations that require more complex nursing care.

*It’s very detrimental to quality (...) especially if it’s a more serious patient, I think it interferes with quality and we end up taking risks, because the number of patients remains the same, but for a smaller number of employees* (FGN3). 

*(...) it can have an impact on the quality of care (...) it can increase absenteeism due to injuries. This affects many things, as we’ve already said: organization, quality, risks to patients and staff (...)* (FGN6). 

*(...) when a lot of patients arrive, I end up doing the minimum for each one (...) (FGNT3).*

There are omissions and/or delays in the provision of care, including risks in medication assistance, due to the various moving of patients during the shift. Planning is compromised or activities are completed hastily or professionals stay beyond their working hours.

*(...) I’m very attentive to all these transfers, documentation, flow, various things, that I end up failing to provide care to other patients as I should (...) (FGN5).*

*(...) when we have a lot of admissions, transfers, things start to slow down in the sector, everything gets slower, patients are waiting for things that sometimes they shouldn’t be (...) (FGN7).*

*(...) leaving the ward compromises medication schedules, care, bathing, dressing and all the activities (...) (FGNT8).*

*(...) I also stay after hours to finish papers and systems* (FGNA3). 

#### Nursing (over)workload

The physical and emotional exhaustion of the team in the face of the unfavorable conditions that permeate their work and the dynamics of moving patients was evident.

*(...) I have to know how to deal with it, but it’s very stressful (...) it bothers me a lot (...). As a nurse, I often get nervous (...) sometimes our psyches get really messed up (FGN3).*

*(...) this is very stressful for professionals, an excessive amount of work, physical and psychological exhaustion which can facilitate errors (FGN12).*

*(...) we all get more stressed too, it’s not nice to work like this, in a rush (...) (FGNA1).*

*(...) we have a lot of patients under our responsibility (...) the day gets very tight and dangerous, some days I’m at my physical and emotional limit (FGNT4).*

In addition, the nursing staff repeatedly mentioned the time factor and estimated that the actions of admitting, transferring and discharging patients in a six-hour shift ranged from 10-15 minutes (FGNA2) to 2-3 hours (FGN1, FGN2, FGN5, FGN6 and FGN11). Most professionals reported 1-2 hours (FGN3, FGN9, FGNT3, FGNT6, FGNA3, FGNT7) dedicated to moving patients. One participant estimated 2-5 hours (FGN12) and three reported 6 hours (FGN8, FGNT2 and FGNT8) dedicated to these activities in a 12-hour shift.

A significant amount of the nursing workday is spent moving patients and, according to the reports, there is still a lack of quality of care and occupational safety.

## Discussion

This study made it possible to recognize factors that are both favorable and unfavorable to professional nursing practice when dealing with the moving of patients in and out of hospital units. The positive aspects were the quantitative and qualitative supply of professionals and resources, collaborative work and the organization of these processes; the adverse aspects were the volume and unpredictability of occurrences, the criticality of inpatients and the lack of staff to meet the demands of care.

 These changes, in the light of the Transitions Theory ^(^ ^1^ ^)^ , represent multidimensional events with vulnerabilities depending on the processes and outcomes of interpersonal relationships with the environment. Nursing interventions can have a positive impact on these transitions. It is therefore important to understand the flow of patients in the units, identifying critical points in order to plan care and define strategies to improve practice. 

 Several studies corroborate the problem of staff shortages ^(5,20-21)^ and other resources ^(5,22-23)^ in the transition of in-hospital care. Patients with more complex care needs are also staying in hospitalization units in other settings in the country ^(^ ^24^ ^-^ ^26^ ^)^ . This demand overloads the nursing team. In Chile, there was evidence of unsafe care and a higher risk of mortality for each patient added to the proportion of nurses ^(^ ^11^ ^)^ . 

 This relationship between supply and demand for care involves dedicated time, physical and cognitive effort and reflects the nursing workload ^(^ ^27^ ^)^ . International benchmarks for nursing interventions show variations of 15 minutes or less for transfers, 16 to 30 minutes for admissions and 46 to 60 minutes to be dedicated to patient discharge. In Brazil, researchers found an average of 19.3 minutes for patient transfers ^(^ ^12^ ^)^ and 13.1 minutes for admissions ^(^ ^13^ ^)^ . In this study, the estimated time spent on interventions throughout the shift (admissions, discharges and transfers) ranged from 10 minutes to 3 hours. In six hours of work, most of the staff reported spending between one- and two-hours moving patients. 

 In addition to time, the professionals also mentioned physical and emotional strain. In this sense, this study reinforces the connection between nursing overload and patient transfers, especially when they occur simultaneously ^(^ ^9^ ^,^ ^21^ ^)^ . It is worth mentioning that, in addition to the transition of care during admissions, discharges and transfers, other types of transition are daily occurrences in nursing care and may require concomitant interventions ^(^ ^1^ ^)^ . Favorable working conditions can minimize the risks of overload and unsafe care, contributing to the retention of nursing professionals ^(^ ^1^ ^,^ ^28^ ^)^ . 

 In addition to organizational aspects, personal conditions interfere with the delivery of care in the various transitions or transfers of patients. As a social practice, the organization of nursing work depends on individual performance and teamwork to develop skills, new functions and relationships in the dynamics of patient care ^(^ ^1^ ^)^ . 

 Collaboration between professionals, as highlighted by the participants in this study, brings benefits to patients, teams and health services. Researchers highlight interprofessional practice and improved communication ^(^ ^3^ ^,^ ^5^ ^)^ using structured tools and/or checklists, as well as other strategies, for the effective transition of care for hospitalized patients ^(^ ^29^ ^)^ , which corroborates the findings of this investigation. In a North American service, 66% of professionals confirmed better care and early identification of risks through the use of these tools ^(^ ^30^ ^)^ . On the other hand, in Spain, 30% of the incidents related to safety in the intra-hospital transfer of patients were due to a lack of personnel, organizational and communication failures on the part of the teams ^(^ ^31^ ^)^ . 

 The nursing team also emphasized communicating admissions, transfers and discharges in advance in order to plan interventions prior to the situation of change, thus qualifying the work carried out. Being aware of the necessary changes is a defining characteristic of transitions and impacts the experience of the patient and the nursing staff ^(^ ^1^ ^)^ . 

 Unlike another study ^(^ ^3^ ^)^ , electronic nursing records and the priorities for occupying beds in the units were not mentioned by the participants as favorable or unfavorable aspects for the transfer of patients and the delivery of care. The management of moving patient has been strategic in optimizing the installed capacity of institutions and qualifying care, but it is still a complex and challenging process ^(^ ^32^ ^)^ . The Transitions Theory underpins this complexity from the perspective of the patient and the nurse, emphasizing the uncertainties, imbalances and competences to be developed in these moving of patients ^(^ ^1^ ^)^ . 

 Higher patient turnover in surgical clinics ^(^ ^7^ ^)^ , more discharges in the morning and transfers between units in the afternoon ^(^ ^12^ ^)^ have been shown in previous studies. In addition to the volume of transfers, N, NT and NA emphasized the time at which they occur, i.e. at the end of the work shift, potentializing care risks and professional overload. Further research could explore this condition more fully. 

 In this study, nurses approached the results of the moving of patients in a specific way, i.e. considering only the moment in the context of hospitalization. This means that the skills and behaviors necessary for patients to manage new situations in the course ^(^ ^1^ ^)^ have not been elucidated. 

 Ensuring resources and information for continuity of care in transition situations is essential ^(^ ^2^ ^)^ . This research confirms these findings and highlights the necessary components for healthy transitions ^(^ ^1^ ^)^ . It was not possible, however, to identify nursing interventions supported in the context of change, but it was possible to recognize that the structure, methods and organization of nursing work need to be improved in order to produce safe moving of patients. 

 In this way, contributions are identified for clinical nurses and managers to dimension and allocate professionals and resources, also taking into account the transfer of patients in and out of the units. The findings also provide tools for evaluating, reviewing and/or systematizing transition processes, encouraging educational actions and the development of new research. It also provokes discussions about caring for patients in intra-hospital transitions based on a Nursing Theory ^(^ ^1^ ^)^ , an innovative aspect of this study. 

This research was limited to the perception of some nursing professionals from specific inpatient units in a hospital practice context that may differ from other realities. Other health professionals or nursing workers from other units may have different perspectives. It is recommended that this research be replicated in other settings, including the perception of patients/families and nursing managers, in order to support decision-making and the improvement of processes.

## Conclusion

The practice of nursing professionals is influenced by structural factors, methods and work organization when they experience admissions, transfers and discharges of patients in the unit. The frequency of these moving of patients the fact that they occur simultaneously and at the end of the shift, as well as unforeseen demands and the complexity of care, tend to have negative repercussions on the delivery of care. It also requires most of the team to dedicate around one to two hours to the work shift.

However, when units have adequate staff and resources/services, systematized processes and collaborative teams, transitions can become smoother, favoring nursing actions and avoiding risks of omission, delay and work overload. It thus provides nurses and managers with tools to implement strategies to minimize problems and improve care in the flow of patients in and out of hospital.
